# Two Mutations in Surfactant Protein C Gene Associated with Neonatal Respiratory Distress

**DOI:** 10.1155/2015/591783

**Published:** 2015-04-27

**Authors:** Anna Tarocco, Elisa Ballardini, Maria Raffaella Contiero, Giampaolo Garani, Silvia Fanaro

**Affiliations:** ^1^Pediatric Section, University Hospital S. Anna, Via Aldo Moro 8, 44124 Ferrara, Italy; ^2^Neonatal Intensive Care Unit and Neonatology, University Hospital S. Anna, Ferrara, Italy

## Abstract

Multiple mutations of surfactant genes causing surfactant dysfunction have been described. Surfactant protein C (SP-C) deficiency is associated with variable clinical manifestations ranging from neonatal respiratory distress syndrome to lethal lung disease. We present an extremely low birth weight male infant with an unusual course of respiratory distress syndrome associated with two mutations in the SFTPC gene: C43-7G>A and 12T>A. He required mechanical ventilation for 26 days and was treated with 5 subsequent doses of surfactant with temporary and short-term efficacy. He was discharged at 37 weeks of postconceptional age without any respiratory support. During the first 16 months of life he developed five respiratory infections that did not require hospitalization. *Conclusion*. This mild course in our patient with two mutations is peculiar because the outcome in patients with a single SFTPC mutation is usually poor.

## 1. Introduction

Genetic disorders of lung surfactant proteins determine abnormal surfactant production and function. Several mutations of surfactant genes responsible for a wide range of phenotypical manifestations, from neonatal respiratory distress to adult chronic lung disease, have been described [1]. Pulmonary surfactant is composed of a lipid mixture and specific proteins. ABCA3 and SP-B are important to absorb surfactant phospholipids into specialized secretory organelles; SP-C and SP-B are required for absorption of the secreted phospholipids into the alveolar surface. SP-C deficiency is a rare autosomal dominant condition associated with interstitial lung disease in children and adults with a variable clinical course [2].

## 2. Case Report

**Figure 1:**
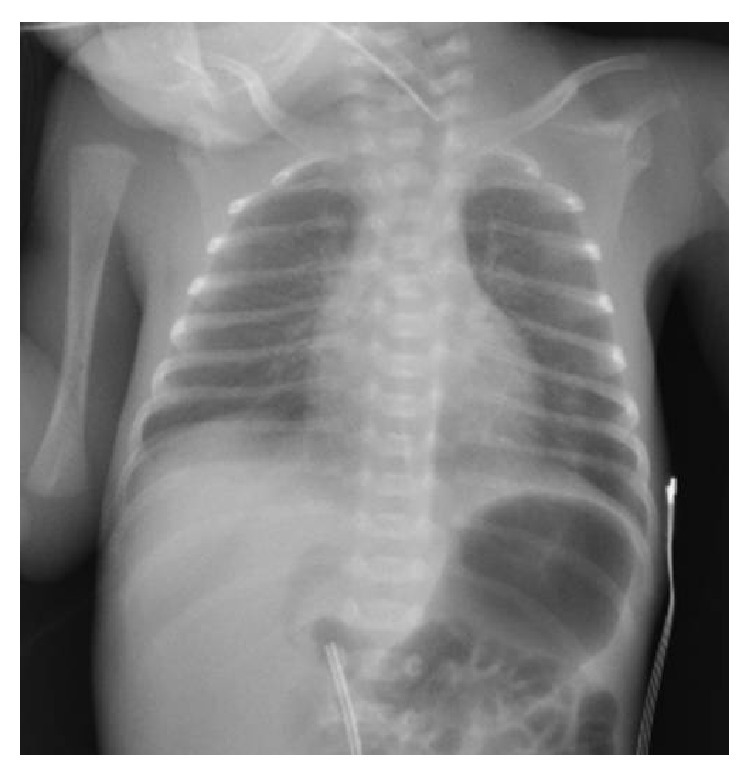
Chest radiograph (first day of life) revealing diffuse haziness of both lungs.

**Figure 2:**
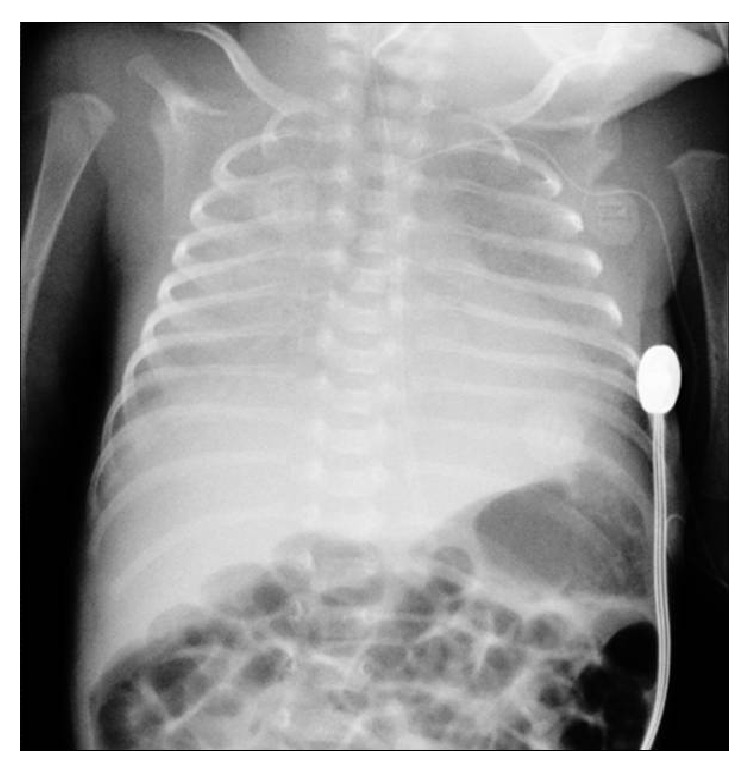
Chest radiograph (20 days of life) showing bilateral diffuse alveolar opacities.

An extremely low birth weight male infant was born at 27th week of gestational age by emergency Caesarean section for onset of labor. Parents were both of African ethnicity (Senegal), not consanguineous, and without family history of note. At birth he required neonatal resuscitation with positive pressure ventilation and intubation. Apgar score was 2-5-8 at 1, 5, and 10 minutes, respectively. During the first hours of life the infant showed a progressive increase of respiratory distress with increased oxygen requirement (up to 60%). The chest radiograph revealed a diffuse haziness of both lungs as shown in Figure 1. The administration of porcine surfactant (200 mg/kg Curosurf, Chiesi) improved ventilatory parameters and allowed extubation. The infant was maintained with continuous positive airway pressure until day 3 of life without supplemental oxygen. On day 4 the physical examination revealed tachypnea, chest retractions, and episodes of apnea requiring reintubation. Mechanical ventilation was then continued for 26 days. During this period he was treated with five subsequent doses of surfactant (100 mg/kg/dose) with temporary efficacy so that three attempts at extubation failed. Chest radiographs showed bilateral diffuse alveolar opacities due to persistent diffuse pulmonary infiltrates (Figure 2). Abundant white fluid tracheal aspirates (repeatedly reported as negative for bacteria and fungi) were documented. The patient was treated with four different antibiotics, fluconazole, and corticosteroids (7-day low-dose treatment with dexamethasone: 0.1 mg/kg/day for 5 days followed by 0.05 mg/kg/day for 2 days) without significant efficacy. On the basis of the clinical response to surfactant, radiographic findings, and white tracheal secretions, surfactant protein deficiency was suspected. Tracheal aspirates and blood for molecular typing for surfactant protein were analyzed: DNA sequencing showed two mutations in the SFTPC gene (C43-7G>A and 12T>A). Parents refused to be examined. The infant was maintained on high flow nasal cannula with oxygen until the 59th day of life. He was discharged at 73 days of life with a weight of 2,700 g without any respiratory support or supplemental oxygen.

During the first 16 months of life the infant developed 5 episodes of bronchiolitis, never requiring hospitalization.

## 3. Discussion

Surfactant, which is secreted by type II alveolar cells, reduces the surface tension maintaining the stability of the lungs. Multiple mutations of the gene of SP-C have been identified, which cause dysfunction of surfactant metabolism and are associated with interstitial lung disease. Nogee et al. described the first case in 2001 [3]. They reported a female term infant with a heterozygous mutation in SFTPC who developed severe respiratory insufficiency at 6 weeks of age. Thomas et al. in 2002 described a large family with variable phenotypic expressions of interstitial lung disease [4]. Three members had been previously described by Donohue et al. in 1959 [5] and Young in 1966 [6] as having a lethal manifestation of pulmonary fibrosis called “fibrocystic pulmonary dysplasia.” Brasch et al. published a case of lethal severe respiratory insufficiency in a 13-month-old baby associated with de novo missense mutation of SFPTC [7]. Tredano et al. published another case of full term baby with early onset of respiratory distress. He progressively developed respiratory distress at 1 month of age, with recurrent bronchitis and dyspnea requiring oxygen supplementation [8]. Soraisham et al. in 2006 reported a case of a term newborn with an unusual presentation and course of lung disease due to single novel mutation in SFTPC gene [9]. Turcu et al. in 2013 described 7 cases of single SP-C gene mutation diagnosed from 3 months of age to 10 years. In two of them symptoms were present at birth; the other children presented later chronic cough, failure to thrive, or oxygen dependency. One of them died at 16 days of life [1]. Recently, Van Hoorn et al. presented a successful weaning from mechanical ventilation in a term newborn with SP-C mutation with severe neonatal respiratory distress treated with methylprednisolone pulse therapy and oral prednisolone [10].

The pathophysiology of this disorder involves intracellular accumulation of a structurally defective SP-C protein. In addition the activity of produced surfactant may be defective [9]. On the basis of the few cases reported, it is evident that clinical manifestations are variable and that the onset ranges from the neonatal period to adulthood. Surfactant protein C deficiency varies from mild temporary tachypnea to lethal respiratory failure due to idiopathic pulmonary fibrosis [11].

At present, no medical therapy for the SFTPC mutation is available. Lung transplantation may be an option in case of severe irreversible respiratory failure. In our report, two mutations of the SFTPC gene were identified. The first one, c43-7G>A, has already been described by Lawson et al. and is associated with idiopathic pulmonary fibrosis [11]. The second, c12T>A, has been reported in dbSNP as a rare variant of the SP-C gene (rs200469074; available at http://www.ncbi.nlm.nih.gov/projects/SNP/). In our patient respiratory distress syndrome resulted from a combination of both prematurity and surfactant abnormality, which accounted for prolonged ventilatory support and for the need of repeated surfactant doses and corticosteroid treatment. At the age of 16 months he is in good clinical condition without any respiratory support and has a normal nutritional status and neurodevelopment. He has developed five respiratory infections that have not required hospitalization. This clinical case highlights the need for molecular and genetic characterization of the surfactant gene especially in preterm patients, when the respiratory distress syndrome has an unusually prolonged course. Our case of two mutations is remarkable since, differently from most cases with even single SFTPC mutations, it was characterized by a particularly mild clinical presentation. There are no reports on the clinical effect of these associated mutations and we may speculate that the c12T>A SP-C gene variant may have no pathogenic effect.
