# Corrigendum to “Two Mutations in Surfactant Protein C Gene Associated with Neonatal Respiratory Distress”

**DOI:** 10.1155/2015/592053

**Published:** 2015-10-19

**Authors:** Anna Tarocco, Elisa Ballardini, Maria Raffaella Contiero, Giampaolo Garani, Silvia Fanaro

**Affiliations:** ^1^Pediatric Section, University Hospital S. Anna, Via Aldo Moro 8, 44124 Ferrara, Italy; ^2^Neonatal Intensive Care Unit and Neonatology, University Hospital S. Anna, Ferrara, Italy

In the paper entitled “Two Mutations in Surfactant Protein C Gene Associated with Neonatal Respiratory Distress” [1], we wrote the following in Section 2 “Tracheal aspirates and blood for molecular typing for surfactant protein were analyzed: DNA sequencing showed two mutations in the SFTPC gene (C43-7G>A and 12T>A). Parents refused to be examined.” It has to be corrected as follows.

“Tracheal aspirates and blood for molecular typing for surfactant protein were analyzed: DNA sequencing showed two mutations in the SFTPC gene (C43-7G>A and 12T>A). The analysis was performed by Clinical and Molecular Biology and Cytogenetics Laboratory, S. Raffaele Hospital (Milan, Italy), directed by Professor Maurizio Ferrari.”
